# β-Cryptoxanthin Improves p62 Accumulation and Muscle Atrophy in the Soleus Muscle of Senescence-Accelerated Mouse-Prone 1 Mice

**DOI:** 10.3390/nu12082180

**Published:** 2020-07-22

**Authors:** Mari Noguchi, Tomoya Kitakaze, Yasuyuki Kobayashi, Katsuyuki Mukai, Naoki Harada, Ryoichi Yamaji

**Affiliations:** 1 Division of Applied Life Sciences, Graduate School of Life and Environmental Sciences, Osaka Prefecture University, Sakai, Osaka 599-8531, Japan; eptmarimon@gmail.com (M.N.); kitakaze.0216@gmail.com (T.K.); sxc02024@edu.osakafu-u.ac.jp (Y.K.); harada@biochem.osakafu-u.ac.jp (N.H.); 2 Daicel Corporation, Konan, Minato-ku, Tokyo 108-0075, Japan; kt_mukai@jp.daicel.com

**Keywords:** β-cryptoxanthin, aging, autophagy, p62, carotenoid, skeletal muscle, muscle fiber, soleus muscle, senescence-accelerated mouse

## Abstract

We investigated the effects of β-cryptoxanthin on skeletal muscle atrophy in senescence-accelerated mouse-prone 1 (SAMP1) mice. For 15 weeks, SAMP1 mice were intragastrically administered vehicle or β-cryptoxanthin. At 35 weeks of age, the skeletal muscle mass in SAMP1 mice was reduced compared with that in control senescence-accelerated mouse-resistant 1 (SAMR1) mice. β-cryptoxanthin increased muscle mass with an increase in the size of muscle fibers in the soleus muscle of SAMP1 mice. The expressions of autophagy-related factors such as beclin-1, p62, LC3-I, and LC3-II were increased in the soleus muscle of SAMP1 mice; however, β-cryptoxanthin administration inhibited this increase. Unlike in SAMR1 mice, p62 was punctately distributed throughout the cytosol in the soleus muscle fibers of SAMP1 mice; however, β-cryptoxanthin inhibited this punctate distribution. The cross-sectional area of p62-positive fiber was smaller than that of p62-negative fiber, and the ratio of p62-positive fibers to p62-negative fibers was increased in SAMP1 mice. β-cryptoxanthin decreased this ratio in SAMP1 mice. Furthermore, β-cryptoxanthin decreased the autophagy-related factor expression in murine C2C12 myotube. The autophagy inhibitor bafilomycin A1, but not the proteasome inhibitor MG132, inhibited the β-cryptoxanthin-induced decrease in p62 and LC3-II expressions. These results indicate that β-cryptoxanthin inhibits the p62 accumulation in fibers and improves muscle atrophy in the soleus muscle of SAMP1 mice.

## 1. Introduction

Skeletal muscle comprises muscle fibers that unite to form muscle fascicle. Skeletal muscle mass accounts for approximately 40% of human body weight [1]. The amount of skeletal muscle depends on the size of the muscle fibers. Skeletal muscle has high plasticity, and exercise increases the size of muscle fibers (muscle hypertrophy). In contrast, insufficient exercise due to a sedentary lifestyle, physical inactivity due to long-term bed rest, and insufficient energy due to malnutrition contribute to a decrease in the size of muscle fibers (muscle atrophy) [2]. In addition, the size of muscle fibers decreases with age, resulting in a decrease in skeletal muscle mass. Skeletal muscle contributes not only to motility but also to energy metabolism. Therefore, skeletal muscle atrophy decreases physical activity and increases the risk of developing metabolic syndromes, such as obesity and type 2 diabetes, ultimately leading to a decreased quality of life and increased mortality. Recently, the use of food ingredients, including supplements and natural edible plants, has attracted attention as a promising method for the prevention of skeletal muscle atrophy.

Skeletal muscle mass depends on the amount of protein in the muscle fiber, which is regulated by the balance between protein synthesis and degradation. The mechanistic target of rapamycin (mTOR) signaling pathway is responsible for protein synthesis in the skeletal muscle [3]. In contrast, the ubiquitin–proteasome and autophagy–lysosome systems both play critical roles in the regulation of muscle protein degradation. In the ubiquitin–proteasome system, the ubiquitination process progresses via an enzymatic cascade, including E3 ubiquitin ligases such as atrogin-1 and MuRF-1; the resultant ubiquitinated proteins are degraded by the proteasome [4]. The autophagy–lysosome system is required for removing and eliminating not only aggregated and misfolded proteins but also damaged cellular organelles. The autophagy–lysosome system constitutively occurs in many physiologic and pathophysiologic processes to maintain homeostasis for intracellular recycling and metabolic regulation. However, age-dependent autophagy impairment has been observed in the skeletal muscle of humans [5].

Intracellular quality is regulated by both nonselective and selective autophagy. SQSTM1/p62 is one of the best characterized cargo receptors of selective autophagy [6,7]. p62 directly interacts with light chain 3 (LC-3) on the isolation membrane and selectively recognizes ubiquitinated cargo proteins as a cargo receptor. Thus, p62 mediates sequestration of the ubiquitinated cargo proteins into autophagosome. p62 encapsulated inside the autophagosome is degraded by the lysosomal enzymes. Impairment of autophagy leads to the p62 accumulation [8]. Many recent studies have prompted an increased interest in the potential role of selective autophagy in the aging process. Autophagy is repressed, and the beclin-1 and p62 expressions are increased in the skeletal muscle of aged mice [9,10]. Moreover, the expression levels of p62, LC3-I, and LC3-II are increased, and the ratio of LC3-II to LC3-I is decreased in the skeletal muscle of aged mice [11]. Senescence-accelerated mice show reduced autophagy activity and increased p62 levels [12].

Senescence-accelerated mouse strains are unique models for studying the aging process [13]. Senescence-accelerated mouse-prone 1 (SAMP1) mice, a strain of senescence-accelerated mouse, are characterized by rapid accumulation of senile features and shortened life spans (the median survival time: 9.7 months for SAMP and 16.3 months for their control senescence-resistant series, SAMR). In SAMP1 mice, the skeletal muscle mass decreases with aging [14,15,16]. Therefore, it is interesting to study the prevention and improvement of age-related skeletal muscle atrophy in SAMP1 mice.

β-cryptoxanthin, a type of oxygenated carotenoid, is classified as a provitamin A and has an antioxidant effect similar to β-carotene. β-cryptoxanthin is one of the carotenoids present in circulating human blood [17]. Observational cohort studies in the elderly have suggested that lower plasma carotenoid levels (the sum of α-carotene, β-carotene, β-cryptoxanthin, lutein/zeaxanthin, and lycopene) are associated with a higher risk of decline in muscle strength and walking speed [18,19,20]. These studies suggest that β-cryptoxanthin has a beneficial effect on the age-related decrease in skeletal muscle mass. However, the effect of β-cryptoxanthin on skeletal muscle mass has not yet been determined. In this study, we evaluated the effects of β-cryptoxanthin on the skeletal muscle mass of SAMP1 mice. We report that β-cryptoxanthin increases skeletal muscle mass and improves p62 accumulation in the soleus muscle of SAMP1 mice.

## 2. Materials and Methods

### 2.1. Animals

The Animal Care and Use Committee of Osaka Prefecture University approved the protocols for animal care and use (approval no.: 19–29). Thirteen-week-old male SAMP1 mice and their control SAMR1 mice, a strain of SAMR series, were purchased from Japan SLC, Inc. (Shizuoka, Japan). The mice were individually housed in cages with free access to water and a non-purified diet (CE-2; CLEA Japan, Inc., Tokyo, Japan) until they were 20 weeks old. The initial body weights of all mice were measured. The SAMP1 mice were randomly assigned into two groups (*n* = 8 per group); SAMP1-control group and SAMP1-CX group. SAMR1 mice were termed as SAMR1 group. Mice were fed a purified diet (AIN-93M, in which sucrose was replaced with cornstarch) ad libitum until 35 weeks of age. From 20–35 weeks of age, the SAMP1-CX group was intragastrically administered micellar β-cryptoxanthin (0.5 mg, once daily), whereas the SAMP1-control and SAMR1 groups were orally administered micelles without β-cryptoxanthin as a vehicle control. Micellar β-cryptoxanthin was prepared according to a previously described method [21], except that β-cryptoxanthin was used instead of β-carotene. The mice were kept in conditioned housing with controlled temperature (23 °C ± 2 °C), humidity (60% ± 10%), and lighting (12 h light/dark cycle starting at 8:00) throughout the experiment. At 35 weeks of age, the animals were sacrificed under an anesthesia, and the skeletal muscle tissues were excised.

### 2.2. Cell Culture

Murine myoblast C2C12 cells were purchased from the European Collection of Authenticated Cell Cultures (Salisbury, UK). The cells were cultured and differentiated into myotubes as described previously [22]. Briefly, myoblasts were cultured in Dulbecco’s modified Eagle’s medium supplemented with 10% fetal bovine serum and antibiotics (termed growth medium). To generate myotubes, myoblasts were cultured in Dulbecco’s modified Eagle’s medium supplemented with 2% horse serum and antibiotics (termed differentiation medium) for 6 days. The differentiation medium was replaced at intervals of 48 h.

### 2.3. Preparation of β-cryptoxanthin

We used enzyme-processed Satsuma mandarin (Citrus unshiu Marc.), which is a powder derived from Satsuma mandarin pulp after juice processing, manufactured by Daicel Corporation (Tokyo, Japan). Non-esterified β-cryptoxanthin was purified from enzyme-processed Satsuma mandarin powder according to the method described by Kobori et al. [23] with minor modifications. Briefly, β-cryptoxanthin was extracted from the enzyme-processed Satsuma mandarin powder with hexane. After evaporating the hexane, the extract was sequentially separated into soluble and insoluble portions with hexane, acetone, ethanol, and a hexane:ethanol (4:6) mixture at −30 °C by centrifugation. In each separation treatment, β-cryptoxanthin was concentrated in the hexane-soluble, acetone-soluble, ethanol-insoluble, and hexane:ethanol-insoluble portions. The concentrated hexane:ethanol-insoluble portion was dissolved in t-butyl methyl ether and hydrolyzed with potassium hydroxide in methanol for 4 days at 4 °C. The organic layer was recovered by adding water and concentrated. The concentrate was dispersed in hexane by sonication. The insoluble substance was filtered, collected, and dissolved in chloroform. The soluble substance was then separated in the solvents system chloroform:methanol (98:2) by thin-layer chromatography (PLC silica gel 60; Millipore Co, Billerica, MA, USA). The silica gel containing β-cryptoxanthin was collected, and β-cryptoxanthin was extracted using chloroform:methanol (98:2). The purity of the β-cryptoxanthin obtained was 95% by high-performance liquid chromatography analysis.

### 2.4. Grip Test

The grip strength of both forelimbs was measured using a grip strength meter (GPM-100; Melquest, Toyama, Japan) according to the method described by Takeshita et al. [24]. The grip strength of each mouse was measured five times in succession, and the average value from the data obtained was represented as the individual value for each mouse (*n* = 6–8 per group).

### 2.5. Cross-Sectional Area

Soleus muscles were dissected from the mice, immediately frozen in chilled isopentane and liquid N_2_, and stored at −80 °C until use. The cross-sections (10-µm thick) of soleus muscle were cut in a cryostat (CM1850; Leica, Wetzlar, Germany) at −20 °C. The muscle sections were air-dried and then fixed with 10% formalin. The sections were washed with phosphate-buffered saline (PBS) and incubated with PBS containing 10% normal serum, followed by reaction with rabbit polyclonal anti-laminin antibody (Sigma-Aldrich, St. Louis, MO, USA) diluted with PBS containing 5% normal serum and 0.3% Triton X-100. The immunoreactive sections were incubated with goat anti-rabbit immunoglobulin G (IgG) (Alexa Fluor 488 conjugate) (Life Technologies, Grand Island, NY, USA) diluted with PBS containing 5% normal serum and 0.1% Triton X-100. Immunofluorescent images were obtained with a BIOREVO BZ-9000 microscope (Keyence, Osaka, Japan). The cross-sectional area (CSA) (µm^2^) of the muscle fibers was measured as described previously [25]. The mean fiber CSA was determined from more than 200 fibers per soleus muscle (*n* = 6–8 per group). The distribution of the CSA was plotted as frequency histograms.

### 2.6. Immunofluorescence

The muscle sections were air-dried and then fixed with 10% formalin. The sections were washed with PBS and incubated with PBS containing 10% normal serum and 0.1% Triton X-100, followed by reaction with polyclonal rabbit anti-p62 antibody (Medical & Biological Laboratories Co., Ltd., Aichi, Japan) diluted with PBS containing 5% normal serum and 0.3% Triton X-100. The immunoreactive sections were incubated with Alexa Fluor 488-conjugated anti-rabbit IgG (Life Technologies, Grand Island, NY, USA) diluted with PBS containing 5% normal serum and 0.1% Triton X-100. Immunofluorescent images were obtained with a confocal laser scanning microscope (LSM700; Zeiss, Tokyo, Japan). The images obtained were analyzed with Image J software (version 1.44p; National Institutes of Health, Bethesda, MD, USA). The percentages of cytosolic p62-positive fibers were calculated by analyzing 150–300 fibers from 10 different parts of the cross-section per mouse (*n* = 5–8 per group), and the ratio of the percentage in the SAMR1 group or in the SAMP1-CX group related to that in the SAMP1-control group was represented. The CSA (µm^2^) of p62-positive and p62-negative fiber in the soleus muscle was measured by analyzing all measurable p62-positive fibers and the surrounding p62-negative fibers.

### 2.7. Western Blot Analysis

For in vivo experiments, the skeletal muscle was minced and homogenized in lysis buffer (50 mM Tris-HCl, pH 7.5, containing 150 mM NaCl, 2 mM ethylenediaminetetraacetic acid, 1% Triton X-100, 1% sodium deoxycholate, 0.1% sodium dodecyl sulfate, 1 mM dithiothreitol, 1 mM phenylmethylsulfonyl fluoride, 1 μg/mL aprotinin, 10 μg/mL leupeptin, 1 mM sodium orthovanadate, 10 mM sodium pyrophosphate, 10 mM sodium molybdate, and 25 mM sodium fluoride). For in vitro experiments, myotubes were cultured with vehicle (dimethyl sulfoxide) or 10 µM β-cryptoxanthin in the presence or absence of the proteasome inhibitor MG132 (10 µM) or the autophagy inhibitor bafilomycin A1 (5 µM) for 48 h. The cells were harvested and lysed in a cell lysis buffer. Tissue homogenates and cell lysates were centrifuged at 20,000× *g* for 15 min, and the supernatants were subjected to sodium dodecyl sulfate–polyacrylamide gel electrophoresis, followed by Western blot analysis using the following antibodies: rabbit polyclonal beclin-1 (cell signaling; Danvers, MA, USA), anti-p62, anti-glyceraldehyde-3-phosphate dehydrogenase (GAPDH) [26], and anti-ubiquitin (cell signaling) antibodies; rabbit monoclonal anti-LC3 (clone D3U4C; cell signaling), anti-atrogin-1 (clone EPR9148(2); Abcam; Cambridge, UK), anti-p70S6K (clone 49D7; cell signaling), anti-phospho-p70S6K (clone 108D2; cell signaling), anti-AMPKα (clone 40H9; cell signaling), anti-mTOR (clone 7C10; cell signaling), and anti-phospho-mTOR (clone D9C2; cell signaling) antibodies; mouse monoclonal anti-β-actin (clone 2D4H5; cell signaling) and anti-myosin heavy chain (MyHC) (clone BA-D5 that recognizes MyHC type I; Developmental Studies Hybridoma Bank, University of Iowa, Iowa City, IA, USA) antibodies; and goat polyclonal anti-MuRF-1 antibody (R&D Systems, Minneapolis, MN, USA). Immunoreactive proteins were incubated with horseradish peroxidase-conjugated goat anti-rabbit IgG, goat anti-mouse IgG, or rabbit anti-goat IgG and were reacted with Immobilon Western Chemiluminescent HRP Substrate (Merck Millipore; Burlington, MA, USA) or Chemi-Lumi One Ultra (Nacalai Tesque Inc., Kyoto, Japan), followed by detection using an LAS4000 imaging system (GE Healthcare, Chicago, IL, USA).

### 2.8. Statistical Analysis

Significant differences between the two groups in animal and cell experiments were determined using the Student’s t-test. Two-way analysis of variance with the Tukey–Kramer post hoc test was used for cell experiments that had four groups. Statistical analysis was conducted using the JMP statistical software (version 8.0.1; SAS Institute, Cary, NC, USA). Data are presented as mean ± standard deviation. Differences were considered significant at *p* values of <0.05.

## 3. Results

### 3.1. β-cryptoxanthin Enhances Grip Strength in SAMP1 Mice

**Figure 1:**
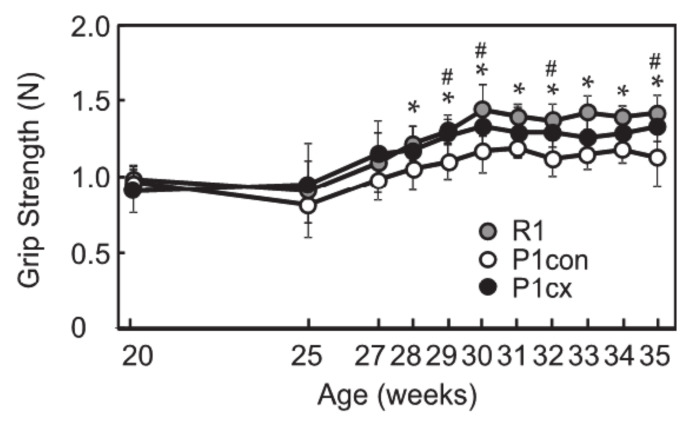
Effects of β-cryptoxanthin on the grip strength of SAMP1 mice. Grip strength of SAMR1 (R1), SAMP1-control (P1con), and SAMP1-CX (P1cx) groups from 20 to 35 weeks of age. Values are represented as mean ± standard deviation. *n* = 6–8 per group. * *p* < 0.05, statistical significance between the SAMR1 and SAMP1-control groups. # *p* < 0.05, statistical significance between the SAMP1-control and SAMP1-CX groups.

To determine the effect of β-cryptoxanthin on skeletal muscle health, β-cryptoxanthin was orally administered to SAMP1 mice from 20 to 35 weeks of age. Cumulative food consumption during the experiment was higher in the SAMP1-control group than in the SAMR1 group and did not significantly differ between the SAMP1-control and the SAMP1-CX groups (Table 1). There was no significant difference in body weight between the SAMR1 and the SAMP1-control groups or between the SAMP1-control and the SAMP1-CX groups at 35 weeks of age. Grip strength was significantly decreased in the SAMP1-control group after 28 weeks old compared with the SAMR1 group (Figure 1). β-cryptoxanthin administration enhanced the grip strength in SAMP1 mice after 29 weeks of age.

### 3.2. β-cryptoxanthin Suppresses Soleus Muscle Atrophy in SAMP1 Mice

**Figure 2:**
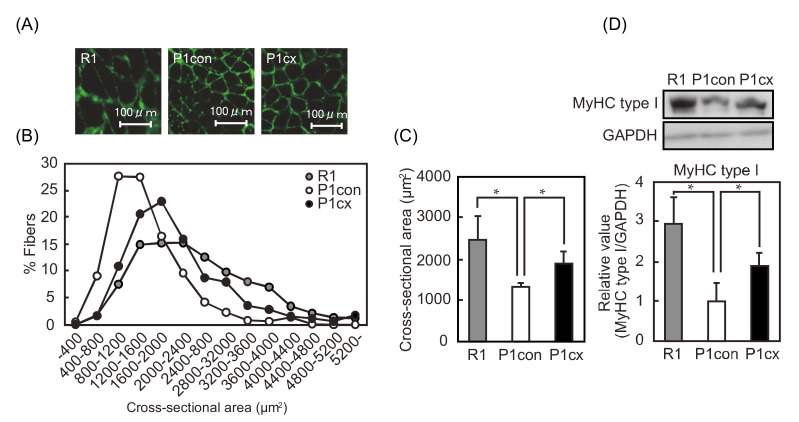
Repression of muscle atrophy by β-cryptoxanthin in the soleus muscle of SAMP1 mice. (**A**) Immunofluorescent staining of laminin in the soleus muscle of the SAMR1 (R1), SAMP1-control (P1con), and SAMP1-CX (P1cx) groups. Scale bar: 100 μm. (**B**) Distribution of soleus muscle fiber cross-sectional area (CSA). (**C**) Average size of the soleus muscle fiber CSA. (**D**) Western blot analyses of MyHC type I and GAPDH. MyHC type I expression was normalized to anti-glyceraldehyde-3-phosphate dehydrogenase (GAPDH) expression. Values are represented as mean ± standard deviation. *n* = 5–8 per group. * *p* < 0.05, statistical significance compared with the SAMP1-control group.

We determined the effects of dietary β-cryptoxanthin on the skeletal muscle mass of SAMP1 mice. Skeletal muscle mass was lower in the quadriceps, extensor digitorum longus (EDL), gastrocnemius, soleus, and plantaris muscles of the SAMP1-control group than in those of the SAMR1 group (Table 2). The ratio of skeletal muscle mass to body weight was lower in the quadriceps, EDL, gastrocnemius, and soleus muscles of the SAMP1-control group than those of the SAMR1 group. β-cryptoxanthin administration to SAMP1 mice increased muscle mass and its ratio to body weight in the soleus muscle. Moreover, we determined the effect of β-cryptoxanthin administration on the size of soleus muscle fiber of SAMP1 mice. The CSA of each muscle fiber in the soleus muscle was fluorescently stained and quantitated (Figure 2A). The frequency distribution of the CSA sifted toward smaller sizes in the SAMP1-control group than in the SAMR1 group and toward larger sizes in the SAMP1-CX group than in the SAMP1-control group (Figure 2B). The mean CSA of the soleus muscle was lower in the SAMP1-control group than in the SAMR1 group and higher in the SAMP1-CX group than in the SAMP1-control group (Figure 2C). The expression level of slow MyHC (type I), a main MyHC type in slow muscle, was lower in the SAMP1-control group than in the SAMR1 group and higher in the SAMP1-CX group than in the SAMP1-control group (Figure 2D).

### 3.3. β-cryptoxanthin Downregulates the Expressions of Autophagy-Related Factors in SAMP1 Mice

**Figure 3:**
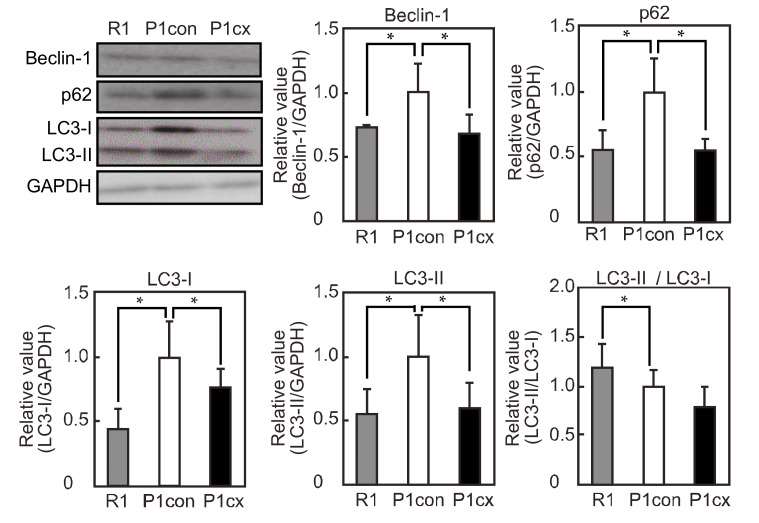
Effects of β-cryptoxanthin on autophagy-related factor expression in the soleus muscle of SAMP1 mice. Western blot analyses of beclin-1, p62, LC3-I, and LC3-II expressions in the SAMR1 (R1), SAMP1-control (P1con), and SAMP1-CX (P1cx) groups. Beclin-1, p62, LC3-I, and LC3-II expressions were normalized to GAPDH expression. Values are represented as mean ± standard deviation. *n* = 5 per group. * *p* < 0.05, statistical significance compared with the SAMP1-control group.

We evaluated the effects of autophagy-related factor expression in the soleus muscle of SAMP1 mice. The expression levels of beclin-1, p62, LC3-I, and LC3-II were higher in the SAMP1-control group than in the SAMR1 group (Figure 3). However, β-cryptoxanthin administration decreased their expression levels in the SAMP1 mice. The ratio of LC3-II to LC3-I was reduced in the SAMP1 group compared with the SAMR1-control group, and dietary β-cryptoxanthin did not affect this ratio in the SAMP1 mice.

### 3.4. β-cryptoxanthin Decreases the p62-Positive Fiber in the Soleus Muscle of SAMP1 Mice

**Figure 4:**
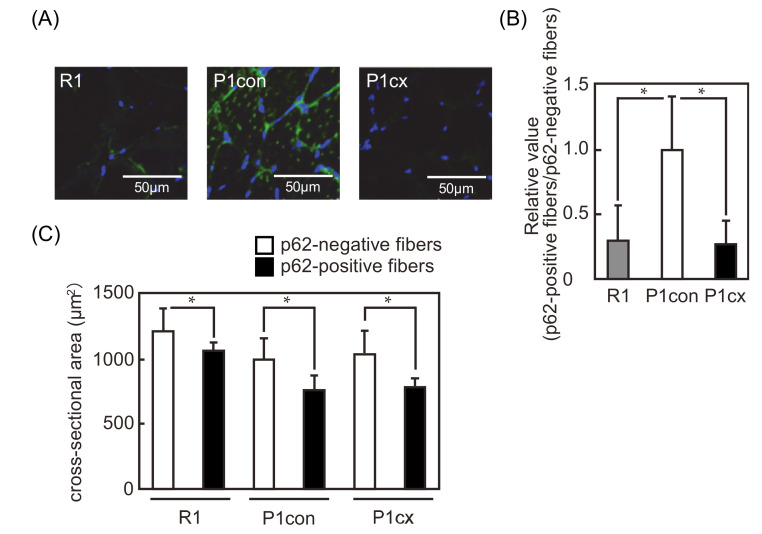
p62-positive fibers in the soleus muscle of β-cryptoxanthin-administered SAMP1 mice. (**A**) Immunofluorescent staining of p62 in the soleus muscle of the SAMR1 (R1), SAMP1-control (P1con), and SAMP1-CX (P1cx) groups. Scale bar: 50 μm. (**B**) Relative value of the ratio of p62-positive fibers to p62-negative fibers in the SAMP1-control group. Values are represented as mean ± standard deviation. *n* = 5–8 per group. * *p* < 0.05, statistical significance compared with the SAMP1-control group. (**C**) CSA of p62-positive fibers (white bar) and p62-negative fibers (black bar). Values are represented as mean ± standard deviation. *n* = 6–8 per group. * *p* < 0.05, statistical significance compared with the CSA of p62-negative fibers.

To evaluate the distribution pattern of p62 in the soleus muscle fibers of the SAMP1 mice and the effect of β-cryptoxanthin on the distribution pattern, the expression pattern of p62 was analyzed by immunofluorescent staining (Figure 4A). The p62 expression was detected in the membrane region rather than in the cytosol of several fibers in the SAMR1 group. Interestingly, p62 was detected in the membrane region and as a punctate distribution throughout the cytosol of several fibers in the SAMP1-control group. However, β-cryptoxanthin inhibited the punctate distribution of p62 throughout the cytosol in the fibers. The ratio of p62-positive fibers to p62-negative fibers was higher in the SAMP1-control group than in the SAMR1 group, and β-cryptoxanthin decreased this ratio in the SAMP1 mice (Figure 4B). Next, we determined the CSA of p62-positive and p62-negative fibers. In the three groups, the mean CSA of p62-positive fibers was smaller than that of p62-negative fibers (Figure 4C).

### 3.5. β-cryptoxanthin Decreases the Ubiquitin Conjugates in the Soleus Muscle of SAMP1 Mice

**Figure 5:**
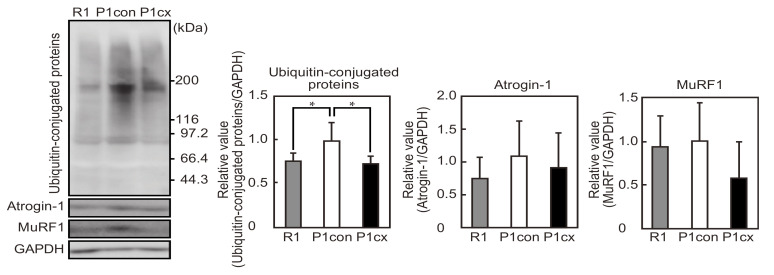
Effects of β-cryptoxanthin on ubiquitin-conjugated proteins and E3 ubiquitin ligase expression in the soleus muscle of SAMP1 mice. Western blot analyses of ubiquitin-conjugated proteins, atrogin-1, and MuRF-1 expression in the SAMR1 (R1), SAMP1-control (P1con), and SAMP1-CX (P1cx) groups. Ubiquitin-conjugated proteins, atrogin-1, and MuRF-1 expressions were normalized to GAPDH expression. Values are represented as mean ± standard deviation. *n* = 5 per group. * *p* < 0.05, statistical significance compared with the SAMP1-control group.

We determined the levels of ubiquitin conjugates and E3 ubiquitin ligases in the soleus muscle of the SAMP1 mice. The levels of ubiquitin conjugates were increased in the SAMP1-control group compared with the SAMR1 group (Figure 5). However, β-cryptoxanthin repressed the increase in SAMP1 mice. In contrast, there was no significant difference in the expression levels of atrogin-1 and MuRF-1 between the SAMR1 and SAMP1-control groups or between the SAMP1-control and SAMP1-CX groups.

### 3.6. β-cryptoxanthin Does Not Influence mTOR Phosphorylation in the Soleus Muscle of SAMP1 Mice

**Figure 6:**
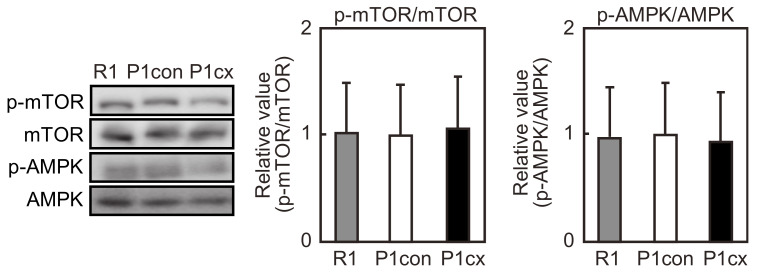
Effects of β-cryptoxanthin on mTOR and AMPK activation in the soleus muscle of SAMP1 mice. Western blot analyses of the expressions of mTOR, AMPK, and their phosphorylated proteins in the SAMR1 (R1), SAMP1-control (P1con), and SAMP1-CX (P1cx) groups. Values are represented as mean ± standard deviation. *n* = 5 per group. * *p* < 0.05, statistical significance compared with the SAMP1-control group.

The ratio of phosphorylation level to the respective total level of mTOR and AMPK proteins in the soleus muscle was not significantly different between the SAMP1-control and SAMR1 groups (Figure 6). β-cryptoxanthin had no influence on the respective ratio in SAMP1 mice.

### 3.7. Bafilomycin A1 Inhibits β-cryptoxanthin-Induced Decrease in p62 Level in C2C12 Myotubes

**Figure 7:**
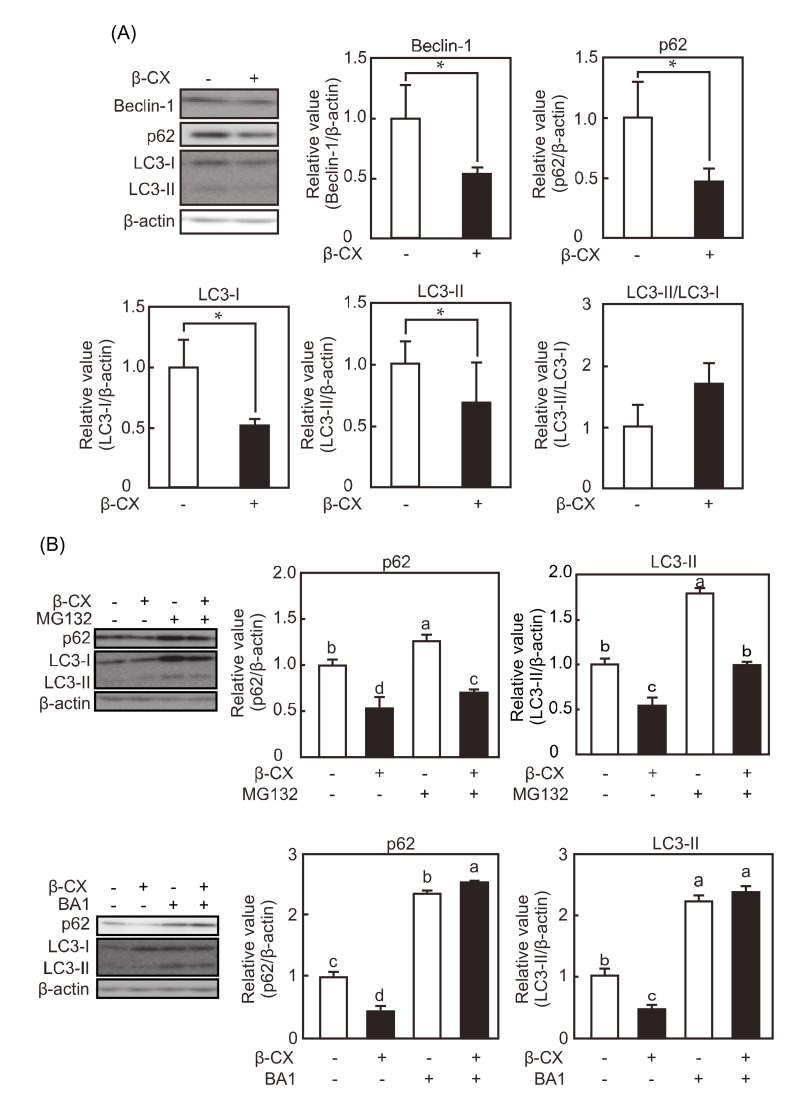
Effects of β-cryptoxanthin and bafilomycin A1 on p62 and LC3-II expression in C2C12 myotubes. (**A**) Western blot analyses of autophagy-related factor expression in β-cryptoxanthin (β-CX)-stimulated C2C12 myotubes. Autophagy-related factor expressions were normalized to β-actin expression. Values are represented as mean ± standard deviation. *n* = 3. Each image is representative of at least three independent experiments. * *p* < 0.05, statistical significance. (**B**) Western blot analyses of p62 and LC3-II expression in β-cryptoxanthin (β-CX)-stimulated C2C12 myotubes in the presence of bafilomycin A1 (BA1) or MG132. p62 and LC3-II expressions were normalized to β-actin expression. Values are represented as mean ± standard deviation. *n* = 3. Each image is representative of at least three independent experiments. Statistical significance *(p* < 0.05) among groups is denoted by different letters.

To evaluate the effects of β-cryptoxanthin on the expressions of autophagy-related factors in vitro, C2C12 myotubes were cultured in the presence or absence of β-cryptoxanthin for 48 h. β-cryptoxanthin decreased the expression levels of beclin-1, p62, LC3-I, and LC3-II and had no influence of the ratio of LC3-II to LC3-I (Figure 7A). Moreover, to evaluate the involvement of autophagy and proteasome in β-cryptoxanthin-induced decrease in p62 and LC3-II levels, C2C12 myotubes were cultured with β-cryptoxanthin in the presence or absence of the autophagy inhibitor bafilomycin A1 and the proteasome inhibitor MG132. Bafilomycin A1 inhibited β-cryptoxanthin-induced decrease in p62 and LC3-II levels, whereas MG132 did not influence their decreases (Figure 7B).

## 4. Discussion

Cells possess diverse mechanisms to eliminate damaged or unnecessary proteins. The autophagy system supports the regulation of intracellular homeostasis and degradation of damaged or dysfunctional proteins and organelles. Inhibition of autophagy in the skeletal muscle results in muscle atrophy and age-dependent muscle weakness [27]. Moreover, the potential role of selective autophagy has been noted in the skeletal muscle, and selective autophagy appears to play a role in the maintenance of skeletal muscle mass during aging [28]. Loss of skeletal muscle mass results in the most recognizable manifestations such as muscle weakness and mobility impairments. Recently, the use of natural dietary supplements has been shown to be a promising strategy to prevent poor skeletal health. In this study, we investigated the effect of β-cryptoxanthin on autophagy in the skeletal muscle.

In 35-week-old SAMP1 mice, the mass of skeletal muscles (quadriceps, tibialis anterior, EDL, gastrocnemius, soleus, and plantaris muscles) was lower than that of age-matched SAMR1 mice. At 28 weeks of age, SAMP1 mice showed decreased mass in only the soleus muscles compared with the SAMR1 mice [25]. These results suggest that in SAMP1 mice, muscle mass was quantitatively affected in slow muscle (e.g., the soleus muscle) rather than in fast muscles (e.g., quadriceps, EDL, gastrocnemius, and plantaris muscles). In the present study, administration of β-cryptoxanthin for 15 weeks (from 20 to 35 weeks) induced an increase in muscle mass accompanied by an increased CSA in the soleus muscle of SAMP1 mice, indicating that β-cryptoxanthin has an atrophy-suppressing effect. Similar to β-cryptoxanthin, β-carotene is a carotenoid detected in human blood [17]. β-Carotene administration suppresses denervation-induced muscle atrophy in the soleus muscle but not in the gastrocnemius muscle of ddY mice [29]. In addition, it reduces thiobarbituric acid-reactive substance (a marker of free radical-mediated lipid peroxidation) levels in the soleus muscle but not in the gastrocnemius muscle. Moreover, dietary β-carotene significantly increases the muscle mass of the soleus muscle but not that of the gastrocnemius, EDL, and plantaris muscles, and raises tetanic force while maintaining specific force (force per CSA) in the soleus muscle [21]. However, in C57BL/6 mice, administration of astaxanthin, which is one of the carotenoids, does not affect mass but increases tetanic force in the EDL muscle [30]. Furthermore, astaxanthin does not affect muscle mass of the plantaris muscle, but it suppresses the degree of immobilization-induced muscle atrophy in rats [31]. These results suggest that β-carotene and β-cryptoxanthin have a more significant quantitative effect on slow muscles, such as the soleus muscle, than that on fast muscles.

The expressions of the autophagy-related factors, such as beclin-1, LC3-I, LC3-II, and p62, were increased in the soleus muscle of SAMP1 mice compared with that of SAMR1 mice, and β-cryptoxanthin suppressed this increase in SAMP1 mice. The increased expressions of these autophagy-related factors in SAMP1 mice were consistent with their expression patterns observed in the gastrocnemius muscle of SAMP8 mice at 36 weeks [32] and 40 weeks of age [12]. The expression of LC3-I, LC3-II, and p62 were higher in the skeletal muscle of aged mice (24–29 months old) than in that of young mice (3.5–7 months old) [11]. The expressions of cytosolic p62 and beclin-1, but not of LC3-I, were increased in the quadriceps muscle of aged C57BL/6 mice (24 months old) compared with young mice (3 months old) [9]. Moreover, beclin-1 and p62 expressions were increased in the gastrocnemius muscle of aged F34/BN rats (24–26 months old) as compared with adult F344/BN rats (7–8 months old), and LC3-I and LC-3II expressions were not affected [33]. Although LC3 expression patterns in aged mice differed among these studies, the increase in p62 expression is a common thread. p62 is a substrate for selective autophagy and is used as an adapter protein (a cargo protein) to selectively degrade ubiquitinated proteins. Because p62 is degraded by lysosomal enzymes after fusion between autophagosomes and lysosomes, selective autophagy disorders result in p62 accumulation. Besides the skeletal muscle of aged animals, autophagy is impaired, and p62 accumulates in the skeletal muscle of muscular dystrophy model mdx mice [34]. It is of interest whether β-cryptoxanthin has a beneficial effect on such skeletal muscle disorders. Moreover, in the present study, β-cryptoxanthin reduced the expression levels of beclin-1, p62, LC3-I, and LC3-II in in vitro experiments, which is consistent with the results obtained from in vivo experiments. The β-cryptoxanthin-induced decreases in p62, LC3-I, and LC3-II expressions were suppressed by bafilomycin A1, an inhibitor of vacuolar-ATPase. Bafilomycin A1 inhibits the fusion between autophagosomes and lysosomes [35]. Taken together, it is suggested that β-cryptoxanthin regulates the expressions of autophagy-related factors by acting on the process of autophagosome fusion or earlier in the autophagy system.

Skeletal muscle has p62-positive and p62-negative fibers, and the ratio of p62-positive fibers to p62-negative fibers increases with age [9]. The CSA of p62-positive fiber is smaller than that of p62-negative fiber. Similarly, in the soleus muscle of SAMP1 mice, the ratio of p62-positive fibers to p62-negative fibers was increased, and the CSA of p62-positive fiber was smaller than that of p62-negative fiber. These results suggest that the muscle atrophy does not uniformly occur in all muscle fibers with age and is induced earlier in p62-positive fibers than in p62-negative fibers. However, dietary β-cryptoxanthin reduced the ratio of p62-positive fibers to p62-negative fibers. These results suggest that β-cryptoxanthin suppresses muscle atrophy by acting on p62-positive fibers in the soleus muscle of SAMP1 mice.

The expression levels of the muscle-specific E3 ubiquitin ligases, atrogin-1 and MuRF-1, did not differ between the soleus muscle of SAMR1 mice and SAMP1 mice. β-cryptoxanthin did not influence the expressions of these E3 ubiquitin ligases. These results suggest that E3 ubiquitin ligases are not involved in soleus muscle atrophy and higher ubiquitin-conjugated protein levels in SAMP1 mice in the present study. In contrast, in the gastrocnemius muscle of SAMP8 mice, the expressions of the E3 ubiquitin ligases were decreased at 36 weeks of age [32] and increased at 40 weeks of age [12], respectively, compared with those in SAMR1 mice. The reason why the present results differ from these previous results remains unclear. However, dietary lysine increases skeletal muscle mass but has no effect on the expressions of the E3 ubiquitin ligases in the gastrocnemius muscle of SAMP8 mice at 36 weeks of age [32]. Moreover, loss-of-function approaches demonstrate that gastrocnemius muscle mass is not significantly different between wild-type and MuRF-1 knockout mice at 23 months of age and is significantly higher in MuRF-1 knockout mice at 33 months of age [36]. However, the gastrocnemius muscle mass in atrogin-1 knockout mice is rather lower than that in wild-type mice at 15 months of age. Thus, MuRF-1, but not atrogin1, may dramatically contribute to the negative regulation of skeletal muscle mass late in aging. The β-cryptoxanthin-induced reduction in p62 and LC3-II expression was not affected by MG132 in in vitro experiments. Taken together, skeletal muscle atrophy during aging in this study appears not to be due to the ubiquitin–proteasome system involving the muscle-specific E3 ubiquitin ligases.

mTOR signaling inhibits autophagy, which accumulates damaged proteins and other unnecessary intracellular molecules [37]. The ratio of phosphorylated mTOR to total mTOR and the ratio of phosphorylated AMPK to total AMPK did not significantly differ between the soleus muscle of SAMR1 mice and that of SAMP1 mice, and β-cryptoxanthin had no influence of their ratios. White et al. [10] evaluated mTOR signaling in the quadriceps muscle of C57BL/6 mice at 4–24 months of age and reported that the p70S6K expression was hyper-phosphorylated at 18 months of age, then decreased. In contrast, the ratio of phosphorylated mTOR to total mTOR decreases in SAMP8 mice at 36 weeks [32] and 40 weeks of age [12]. However, the reason why there is a difference in results between this study and the previous studies remains unknown. Food intake affects the activation of mTOR signaling [10]. The dietary status of mice evaluated for the mTOR signaling may be different between the previous studies and the present study. However, AMPK is activated via phosphorylation, and activated AMPK suppresses mTOR signaling [38]. Thus, in this study, mTOR signaling appears not to contribute to soleus muscle atrophy and β-cryptoxanthin-attenuated soleus muscle atrophy in SAMP1 mice.

Rubicon expression is increased in aged mouse and Rubicon-induced suppression of autophagic activity is one of the signatures of aging [39]. Therefore, to assess whether the myotubes used in in vitro experiments are senescent cells, Rubicon expression was determined at 0, 24, and 48 h because myotubes were cultured with β-cryptoxanthin for 48 h. Rubicon expression remained unchanged during the culture (data not shown). These results indicated that the present in vitro model did not reflect aging of the in vivo model. Although β-cryptoxanthin reduced p62 expression in both in vivo and in vitro experiments, it remains unclear whether the effects of β-cryptoxanthin on p62 expression proceed through the same molecular mechanism in in vitro and in vivo experiments. In order to clarify the effects of β-cryptoxanthin in vivo, we need to establish the in vitro experimental model in which Rubicon expression is increased as one of signatures of aging.

## 5. Conclusions

This study demonstrates that β-cryptoxanthin suppresses the decrease in muscle mass and CSA in the soleus muscle of SAMP1 mice. Results from in vivo experiments indicate that p62-positive fiber has a smaller CSA than p62-negative fiber and that the ratio of p62-positive fiber to p62-negative fiber increases in the soleus muscle of SAMP1 mice and that β-cryptoxanthin suppresses this increase. Results from in vitro experiments suggest that the β-cryptoxanthin-suppressed p62 expression is regulated by autophagy. Taken together, β-cryptoxanthin appears to suppress the decrease in autophagy in the soleus muscle during aging. Therefore, β-cryptoxanthin may be an effective food ingredient to prevent muscle atrophy during aging by restoring or inhibiting the decrease in autophagy activity.

## Figures and Tables

**Table 1:** Food consumption and body weight of mice.

	SAMR1	SAMP1-Control	SAMP1-CX
Initial body weight (g)	37.5 ± 2.7	34.4 ± 5.4	32.8 ± 2.6
Final body weight (g)	39.6 ± 2.2	39.9 ± 0.9	39.4 ± 3.2
Total food consumption (g)	324.1 ± 10.3 *	346.0 ± 20.8	344.5 ± 9.2

Values are represented as mean ± standard deviation. *n* = 6–8 per group. * *p* < 0.05 vs. SAMP1-control. SAMR1, senescence-accelerated mouse-resistant 1; SAMP1, senescence-accelerated mouse-prone 1.

**Table 2:** Skeletal muscle mass of mice.

	SAMR1	SAMP1-Control	SAMP1-CX
Muscle mass (mg)			
Quadriceps	425.3 ± 18.7 *	368.0 ± 4.1	371.5 ± 30.0
Tibialis anterior	105.9 ± 5.7	98.6 ± 10.5	104.3 ± 7.5
EDL	21.5 ± 1.5 *	17.5 ± 2.2	17.4 ± 2.1
Gastrocnemius	273.5 ± 15.4 *	206.5 ± 23.9	204.3 ± 13.5
Soleus	18.4 ± 1.5 *	12.7 ± 1.1	14.2 ± 1.1 *
Plantaris	39.9 ± 15.1	26.0 ± 2.6	26.7 ± 1.9
Muscle mass/body weight (mg/g)			
Quadriceps	10.74 ± 0.37 *	9.63 ± 3.93	9.50 ± 1.30
Tibialis anterior	2.68 ± 0.25	2.57 ± 0.11	2.66 ± 0.32
EDL	0.54 ± 0.04 *	0.46 ± 0.04	0.44 ± 0.06
Gastrocnemius	6.91 ± 0.43 *	5.32 ± 0.52	5.23 ± 0.76
Soleus	0.47 ± 0.04 *	0.33 ± 0.01	0.36 ± 0.03 *
Plantaris	1.01 ± 0.40	0.67 ± 0.06	0.68 ± 0.09

Values are represented as mean ± standard deviation. *n* = 6–8 per group. * *p* < 0.05 vs SAMP1-control. EDL, extensor digitorum longus.
